# Plasma microRNA Array Analysis Identifies Overexpressed miR-19b-3p as a Biomarker of Bipolar Depression Distinguishing From Unipolar Depression

**DOI:** 10.3389/fpsyt.2020.00757

**Published:** 2020-08-11

**Authors:** Yu Chen, Jiabo Shi, Haiyan Liu, Qiang Wang, Xiangxiang Chen, Hao Tang, Rui Yan, Zhijian Yao, Qing Lu

**Affiliations:** ^1^ Department of Psychiatry, The Affifiliated Brain Hospital of Nanjing Medical University, Nanjing Medical University, Nanjing, China; ^2^ Department of Medical Psychology; Nanjing Drum Tower Hospital, Medical School of Nanjing University, Nanjing, China; ^3^ Department of Psychiatry, Nanjing Brain Hospital, Medical School of Nanjing University, Nanjing, China; ^4^ School of Biological Sciences & Medical Engineering, Southeast University, Nanjing, China; ^5^ Child Development and Learning Science, Key Laboratory of Ministry of Education, Nanjing, China

**Keywords:** bipolar disorder, unipolar depression, peripheral miRNAs, biomarker, gene expression

## Abstract

**Objectives:**

The clinical characteristics of bipolar disorder (current major depressive episode) (BD) overlap with unipolar depressive disorder (UD), which makes it difficult to perform an accurate diagnosis. We identified plasma microRNAs (miRNAs) that distinguished BD from UD and explored the relationship between miRNA expression levels and clinical characteristics.

**Methods:**

Total miRNAs from blood plasma from seven UD patients, seven BD patients, and six controls were analyzed. The identified miRNAs were validated in a separate population group. Depression severity and early life adversities were assessed. Bioinformatic analysis was conducted to investigate the target genes that were identified and the pathways associated with the altered miRNAs.

**Results:**

Compared to controls, 42 miRNAs were differentially expressed in patients. miR-19b-3p, miR-3921, and miR-1180-3p were selected to validate the microarray results. Only miR-19b-3p was validated as down-regulated in patients. The primary predicted genes associated with miR-19b-3p were MAPK1, PTEN, and PRKAA1. The most relevant KEGG pathways included mTOR, FoxO, and the PI3-K/Akt signaling pathway. BD patients were more likely to have higher expression levels of miR-19b-3p and more severe childhood trauma experience compared to UD patients.

**Conclusions:**

Plasma miR-19b-3p is a potential non-invasive biomarker that might be useful in distinguishing UD from BD. miR-19b3p was predicted to be involved in the pathway of inflammatory dysregulation associated with experiencing early childhood trauma.

## Introduction

The clinical manifestations of bipolar disorder (current depressive episode) (BD) and unipolar depressive disorder (UD) have a number of similar characteristics. Both disorders present a substantial public health burden due to their high prevalence, recurrence, and degree of disability. Although the theoretical basis of clinical psychiatry has been developed for more than half a century from Kraepelin’s conceptual foundations of mental illness to descriptive symptomatological conception, it still is not easy to clearly distinguish BD from UD. An episode of major depression may occur in the early stage of BD such that the patient is misdiagnosed with UD due to the lack of any history of hypomanic or manic episodes. It is possible for clinicians to change a diagnosis over time when evidence of hypomanic or manic episodes appears. The time-delay gap between the onset and the accurate diagnosis of BD, on average, is approximately five to ten years (1). Delayed diagnosis of BD may lead to a deleterious outcome through the use of antidepressant monotherapy, which would increase the risk of ‘switching’ into hypomanic or manic episodes (2, 3). Approximately 40% to 70% of BD patients have to confront the problem of receiving an appropriate diagnosis as early as possible (4, 5). It is imperative for clinicians to make a precise diagnosis to discern BD from UD. A series of clinical assessments have been administered, including sub-threshold hypomania, atypical depressive symptoms, and other clinical characteristics (6–8). Nonetheless, it is not enough to effectively distinguish the disorders using only these clinical symptoms.

Given the significant heritability of UD and BD, candidate genes for both diseases have been identified (9–11). One genome-wide association study (GWAS) that focused on major depression and included over 135,000 individuals identified 44 loci with genome-wide significance (12). Another GWAS that included over 20,000 BD patients found 30 significant genomic loci (13). However, these GWAS presented poor replication and failed to find shared or distinct genetic markers of affective disorder. One reason for this failure is that psychiatric diseases result from environmental causes as well as predisposing genetic factors. Epigenetic mechanisms indicate the interaction of genetic and environmental factors (G*E interaction). Given the role of non-coding RNAs as post-transcriptional regulators of gene expression, microRNAs are considered to be one of the G*E pathological features of affective disorders. Thus, the use of microRNA expression is of considerable interest to detect and distinguish UD from BD (14–17).

MicroRNAs (miRNAs) are small, endogenously-expressed, non-coding RNAs (~22 nucleotides) that can repress translation to inhibit protein synthesis or promote degradation of target mRNAs with complementary sequences (18). One miRNA can target hundreds of different mRNAs, and multiple miRNAs may regulate a single mRNA. Nearly 70% of mammalian miRNAs are expressed in the brain that generally negatively regulate target gene expression. Emerging studies have demonstrated that miRNAs, which are regarded as “neural communication sculptors,” play an essential role in the proliferation, differentiation, and migration of neurons and participate in the regulation of neuropsychiatric disorders (19, 20). Zhao et al. reported that sevoflurane-induced upregulation of miR-19-3p in neonatal rats post-transcriptionally inhibited protein translation of CCNA2, which contributed to the impairment of learning and memory (21). Clinical studies have reported altered miRNA expression in different brain regions of patients with schizophrenia, bipolar disorders, and major depressive disorder (22, 23).

MiRNAs are also released and circulating in the serum, plasma, and other body fluids. The signatures of circulating miRNAs may be potentially useful non-invasive biomarkers for disease, such as psychiatric disorders. For example, circulating miRNAs were shown to be changed by electroconvulsive shock therapy in psychotic depression (24). Walker et al. identified differential miRNAs (miR-15b, miR-132, and miR-652) in whole-blood samples of bipolar disorder comparing to healthy controls (25). Another study found a set of circulating miRNAs (let-7a-5p, let-7d-5p, let-7f-5p, miR-24-3p, and miR-425-3p) that were specifically altered in major depressive patients, five miRNA transcripts (miR-140-3p, miR-30d-5p, miR-330-5p, miR-378-5p, and miR-21-3p) were specifically altered in bipolar disorder patients, and two miRNAs (miR-330-3p and miR-345-5p) were altered in both diseases (26). Though the potential use of circulating miRNAs for psychiatric disorders screening has emerged, whether circulating miRNAs can be used as biomarkers for distinguishing bipolar depression from unipolar depression is still unclear.

Among the numerous adverse environmental conditions that exist, early childhood trauma, such as emotional abuse or neglect, physical abuse or neglect, and sexual abuse, is the greatest risk factor for the onset and development of depression (27). Patients diagnosed with UD or BD experienced more childhood trauma than healthy subjects (28, 29). Neuroimaging studies have shown that early life adversities were associated with structural and functional abnormalities in specific brain regions that are involved in cognitive processing and emotional regulation (30). Growing evidence supports a direct association between exposure to childhood trauma and elevated levels of inflammatory biomarkers, such as C-reactive protein, interleukin-6, and white blood cell counts (31). The impact of early life adversity and a dysregulated inflammatory profile would persist into adulthood, leading to greater vulnerability to depression (32, 33). It is hypothesized that the activation of immunological processes might be the mediator between childhood trauma and psychopathological outcomes (34).

During the process of adaptation to environmental perturbations by the individual, gene expression is modulated dynamically to optimize responses to stress. Emerging evidence indicates that miRNAs are ideally positioned to coordinate the genomic response to stress (35). Stress-induced alterations in miRNA expression affect multiple biological processes, including neurotransmission, cytokine production, and inflammation. Understanding the role of miRNAs in regulating stress-induced gene expression could have diagnostic benefits in mood disorders. To date, no studies have been conducted that compare the interactions between alterations in miRNA expression and early life adversities in UD and BD patients. The present study used an array to assess genome-wide plasma miRNA expression in UD and BD patients in combination with the assessment of the environmental risk factor of childhood trauma. The results of this study present a novel and comprehensive molecular signature that can contribute to the differential pathogenesis of UD and BD.

## Materials and Methods

### Participants

Patients who were experiencing a major depressive episode were recruited from the Department of Psychiatry of the Affiliated Nanjing Brain Hospital of Nanjing Medical University from 2015 to 2016. The inclusion criteria for patients were as follows. (i) The patients received a diagnosis from a senior psychiatrist of major depressive disorder (unipolar depression, UD) or bipolar disorder (type I or type II) (BD) according to the Structured Clinical Interview for DSM-IV (SCID-IV). (ii) The patients received a 24-item Hamilton Depression Rating Scale (HAMD-24) score equal to or greater than 20. (iii) The age of the patients was between 18 and 55 years. (iv) The patients did not receive any psychotropic medications (including antipsychotics, antidepressants, mood stabilizers, and benzodiazepines) for at least four weeks. The exclusion criteria included the following. (i) The patients were diagnosed with other DSM-IV psychiatric disorders. (ii) The patients had a history of severe head injury. (iii) The patients were diagnosed with any neurological diseases or severe physical diseases, as evaluated by laboratory tests or personal history. (iv) The patients had a history of alcohol or substance dependence or abuse. (v) The patients were pregnant or lactating. All patients who were included in the study were followed up every six months until December 2018. The different types of early life stress were assessed among all patients when they entered the study using the Childhood Trauma Questionnaire (CTQ) (36). At every follow-up time point, the UD patients were administered the 32-item hypomania checklist (HCL-32) to screen for hypomania symptoms (37). If the HCL-32 score was greater than 14 points, the patient’s diagnosis was switched to BD, and the patient was excluded from the study.

The healthy control group included individuals who were matched to the patients with respect to age, gender, and education. The control subjects were recruited from communities in Nanjing from 2015 to 2016 and were screened using the Mini International Neuropsychiatric Interview (MINI) (38). Healthy controls were excluded if they had any history of psychiatric disorders or had any family history of mental disorders in their first-degree relatives.

All subjects were genetically unrelated, ethnic Han Chinese, with at least six years of education. Each subject donated 5 ml of venous blood at the time of their recruitment. A two-phase study was designed. First, in the screening phase, we performed peripheral miRNA profiling using Affymetrix chips for UD patients, BD patients, and healthy controls who were randomly selected from the sample set. In the second, independent validation phase, we examined the expression levels of identified miRNAs in all participants and analyzed the results of the miRNA arrays using the Gene Ontology and KEGG Pathway assays. Finally, we assessed the shared and distinct correlations between the expression of selected miRNAs and childhood traumatic experiences in UD and BD patients.

This study was approved by the institutional review board of the Affiliated Nanjing Brain Hospital of Nanjing Medical University, and written informed consent was obtained from each participant.

### Plasma Preparation and RNA Isolation

The plasma was separated from venous blood within 24h after collection by centrifugation at 12,000 r.p.m. for 15 min. The supernatant from the plasma samples was stored in 300 µl aliquots at −80°C in RNase-free microtubes until it was used for miRNA extraction. A modified method was utilized to isolate total RNA. Briefly, Trizol reagent (Invitrogen, Carlsbad, CA, USA) was used to break down cells and cellular components in the plasma, and total RNA was extracted and purified using a miRNeasy Serum/Plasma Kit (Qiagen, Valencia, CA, USA) according to the manufacturer’s instructions. RNA quality and quantity were evaluated using a NanoDrop ND-1000 spectrophotometer (Thermo Fisher Scientific, Waltham, MA, USA).

### miRNA Array

During the screening phase, a volume corresponding to 500 ng of total RNA from each blood sample was processed using a FlashTag Biotin HSR RNA Labeling kit (Affymetrix, Santa Clara, CA, USA), following the manufacturer’s protocol. The RNA was subsequently hybridized onto Affymetrix GeneChip miRNA 4.0 Arrays that each contained 2,578 human miRNA sequences (Affymetrix, Santa Clara, CA, USA). The GeneChip miRNA 4.0 Arrays were washed and stained using aFluidics station 450 and a GeneChip Scanner 3000 7G (Affymetrix, Santa Clara, CA, USA), respectively.

### qRT-PCR Validation

At the validation phase, quantitative real-time PCR (qRT-PCR) was performed using Taqman microRNA probes (Applied Biosystems Inc, CA, USA) to confirm the candidate miRNAs identified on the microarrays. Total RNA was reverse transcribed to complementary DNA using a miRNA 1st Strand cDNA Synthesis Kit, stem-loop RT primers (Vazyme, Nanjing, China), and the GeneAmp 9700 PCR System (Thermo Scientific, MA, USA). The reactions began in a 384-well optical plate at 95°C for 5 minutes, followed by 40 cycles of 95°C for 10 seconds and 60°C for 30 seconds. The quantitative detection of the miRNAs was performed using the miRNA Universal SYBR qPCR Master Mix (Vazyme, Nanjing, China) and implemented on the Agilent AriaMx platform (Agilent Technologies, Palo Alto, CA, USA). All reactions, including no-template controls, were performed in triplicate. To calculate the relative expression levels of the target miRNAs, U6 was used as the control miRNA for plasma samples (the sequences of the primers listed in **Supplemental data 1**).

### Data Analysis

All statistical analyses were performed using R (version 3.5.0) and SPSS (version 24.0). Demographic and clinical characteristics among the three groups were compared using χ2 tests, the Student’s t-test, or one-way ANOVA.

CEL-files of raw data were produced using Affymetrix GeneChip Command Console Software, Version 4.0 (Affymetrix, Santa Clara, CA, USA). Arrays were normalized using quantile normalization, and the probe values were log2 transformed. All data have been submitted to the GEO repository (code number: GSE152267).

We utilized one-way ANOVA to detect differently expressed miRNAs among all three groups, and miRNAs were chosen as candidates for further confirmation by individual qRT-PCR according to the following three criteria: (i) *p-*value <0.05 for the ANOVA; (ii) assessed using Fisher’s Least Significant Difference test as the Post Hoc test for multiple comparisons; (iii) fold changes (FCs) ≥3/2 or ≤2/3 between every two groups (BD vs. controls, UD vs. controls, and BD vs. UD).

Concerning qRT-PCR validation, the Ct values were normalized according to the delta Ct (ΔCt) method based on the internal reference, U6. The relative expression levels of target miRNAs were calculated using 2^-ΔΔCT^, which were analyzed using the Student’s t-test for independent samples (39). Binary logistic regression was applied to determine correlations between miRNA expression levels and childhood traumatic variables. A *p*-value<0.05 was statistically significant for all analyses.

### Target Gene Prediction and Pathway Analysis

Bioinformatic analysis (Genminix Informatics Ltd., Shanghai, China) was performed for the miRNAs expressed in significant amounts. TargetScan (http://www.targetscan.org/) and miRanda (http://www.microrna.org/microrna/hom.do) were used to predict target genes for the validated candidate miRNAs, and only genes predicted by both databases were retained. To identify the potential biological mechanism of differentially expressed miRNAs among the BD, UD, and healthy controls, we performed gene ontology (GO, http://www.geneontology.org/) and Kyoto Encyclopedia of Genes and Genomes (KEGG, http://www.genome.jp/kegg/) pathway enrichment analysis with the David web application (https://david.ncifcrf.gov). Fisher’s exact test was employed to determine whether a gene set was enriched for a specific gene using GO terms or KEGG pathways compared to the background information. The p-value was corrected for false discovery rates (FDR). GO terms with a p-value<0.01 and a KEGG pathway with a *p-*value<0.05 were considered significant. The miRNA-mRNA-gene network of miR-19b-3p was obtained by using Cytoscape (Version 2.8.2).

## Results

### Demographic and Clinical Characteristics of the Subjects

All subjects were followed up every six months to confirm whether they experienced any hypomania or mania episodes until December 2018. During three years of follow up, six UD patients (two males and four females, with a mean age of 26.2 ± 10.0 years) experienced a manic episode that lasted more than seven days. These six patients were excluded from the study since their diagnosis was switched from UD to BD. Thus, 32 UD patients and 27 BD patients were included in the final analysis (see **Table 1**). There was no statistical difference between these two groups regarding age, gender, education, or family history (*p*>0.05). The UD group recruited more first-episode patients compared to the BD group, while patients in the BD group had a longer duration of illness than the UD group. Clinical assessments showed that there were no significant differences in the total scores for HAMD between the two diagnostic groups. At the same time, the UD patients had higher anxiety or somatization scores (*p*<0.05) and sleep disturbance scores (*p*<0.01) compared to BD patients.

**Table 1:** Demographic and clinical characteristics of unipolar depression, bipolar depression, and healthy controls.

Variables	Total participants			Significance	
	UD (n = 32) Mean (SD)	BD (n = 27) Mean (SD)	HC (n = 18) Mean (SD)	*p* (UD vs. BD)	*p* (three groups)
**Age (years)**	35.06 ± 8.20	31.06 ± 9.97	32.56 ± 6.64	0.114	0.192
**Age range (years)**	19~51	18~54	21~46		
**Female (n(%))**	21 (66.7%)	19 (70.4%)	8 (44.4%)	0.698	0.188
**Education (years)**	13.53 ± 3.39	13.74 ± 2.64	13.67 ± 3.07	0.957	0.965
**Duration of illness (months)**	17.44 ± 17.37	36.15 ± 42.37		0.026*	
**Family history (n(%))**	8 (25.0%)	8 (29.6%)		0.690	
**Depression for first episode (n(%))**	29 (90.6%)	15(80.0%)		0.002**	
**Clinical assessment**					
**Total score of HAM-D_24_**	31.38 ± 6.04	28.63 ± 5.20		0.069	
**Subscore of HAM-D_24_**					
** Anxiety/somatization**	8.22 ± 3.20	6.33 ± 2.48		0.016*	
** Cognitive disturbance**	3.97 ± 1.98	4.37 ± 1.76		0.417	
** Retardation**	7.84 ± 1.55	8.26 ± 1.89		0.357	
** Hopelessness**	5.22 ± 1.95	5.00 ± 2.11		0.681	
** Sleep disturbance**	4.63 ± 1.29	3.67 ± 1.71		0.007**	
** Weight loss**	1.19 ± 0.93	0.52 ± 0.89		0.719	
** Circadian fluctuation**	0.31 ± 0.59	0.26 ± 0.53		0.603	
**Total score of CTQ**	50.75 ± 11.7	54.85 ± 14.11	44.67 ± 3.76	0.018*	
**Subtypes of CTQ**					
** Emotional abuse**	8.94 ± 3.88	10.30 ± 5.53	6.78 ± 1.11	0.025*	
** Physical abuse**	6.47 ± 2.26	6.89 ± 2.93	4.94 ± 0.24	0.019*	
** Sexual abuse**	5.72 ± 1.87	5.81 ± 1.67	5.00 ± 0.00	0.195	
** Emotional neglect**	13.38 ± 4.69	15.48 ± 5.32	10.89 ± 2.42	0.005**	
** Physical neglect**	9.19 ± 3.73	9.41 ± 4.12	7.22 ± 2.39	0.110	

UD, unipolar depression; BD, bipolar depression; HC, healthy control; HAM-D24, 24-item Hamilton Depression Scale; CTQ, Childhood Trauma Questionnaire. *p < 0.05, **p < 0.01.

### miRNA Expression Profiles

Seven UD patients, seven BD patients, and six healthy controls were randomly selected from the total sample to undergo microarray chip inspection. There were no differences in the subjects’ demographic characteristics, the overall severity of depression, or childhood traumatic experiences (see **Supplementary data 2**). Total RNA extracted from peripheral venous blood was analyzed using Affymetrix miRNA 4.0 Arrays. The altered miRNA expression in UD and BD patients compared to healthy controls are listed in **Supplementary data 3**. Seventeen miRNAs were down-regulated, and 25 miRNAs were up-regulated (*p*<0.05).

**Figure 1:**
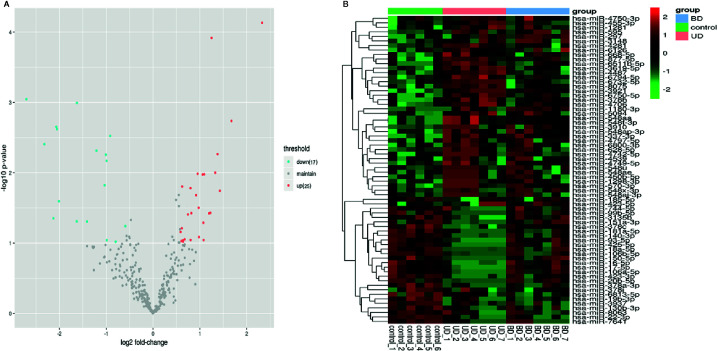
**(A)** Volcano map of differentially expressed miRNAs. The horizontal axis is the logarithm of the fold change value based on 2, and the vertical axis is the negative logarithm of the p value based on 10. Blue dots represent down-regulated miRNAs with statistically significant differences in patients comparing healthy subjects. Red dots represent miRNAs that are up-regulated in patients, and grey dots represent miRNAs that have no significant differences in expression values between the patients and healthy controls. **(B)** Heat map of differentially expressed miRNAs. The abscissa represents the subject names between groups, and the ordinate represents the differentially expressed miRNAs. Differentiated miRNAs are expressed in red with high expression values and those with low expression values are in green.

Hierarchical clustering was carried out for all covered human mature miRNAs and pre-miRNAs, and significant differences in expression values were observed between the patients diagnosed with UD and BD and healthy controls. (**Figure 1**). Following hierarchical clustering, the array results were narrowed down to dysregulated miRNAs with a *p*-value < 0.05 from one-way ANOVA analysis of the three groups and a fold change ≥3/2 or ≤2/3 for all the two-group comparisons. Based on these criteria, three representative miRNAs (miR-19b-3p, miR-3921, and miR-1180-3p) were selected for PCR to validate the microarray results.

### Verification Using Quantitative Real-Time PCR

**Figure 2:**
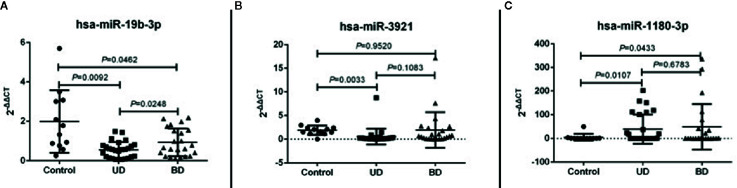
Scatter plot figures show qRT-PCR validation of the differential expression of three miRNAs among three groups (unipolar patients (UD), bipolar patients (BD), and healthy controls (HC) **(A)** miR-19b-3p, **(B)** miR-3921, **(C)** miR-1180-3p). The values of 2-ΔΔCT represent the expressed level of filtered miRNAs. A *p*-value of < 0.05 means statistically significant.

Quantitative real-time PCR (qRT-PCR) was performed to verify the differentially expressed miRNAs in an expanded sample comprised of 32 UD patients and 27 BD patients. Only miRNAs with a fold change ≥3/2 or ≤2/3 and a *p*-value < 0.05 from Student’s t-tests for each two-group comparison were validated. Of these, miR-19b-3p was verified to be down-regulated (see **Figure 2**).

### Microarray-Based Gene Ontology and Signal Pathway Analysis

**Figure 3:**
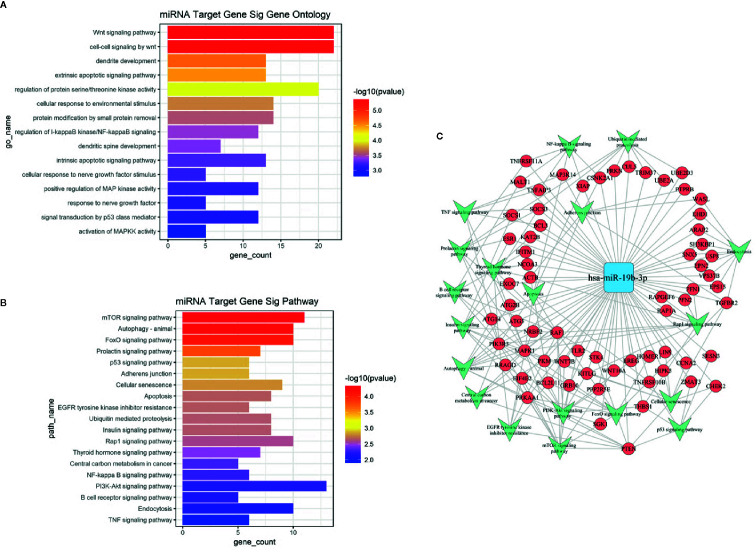
**(A)** GO analysis of target genes predicted by miR-19b-3p. The ordinate is the name of the target gene function, and the abscissa is the negative logarithm of *p* value (-Lg*p*). The larger the -Lg*p* value, the smaller the *p* value and the higher the significance level of the target gene function. **(B)** KEGG signal pathway of target genes predicted by miR-19b-3p. The ordinate is the name of the target gene signal pathway, and the abscissa is the negative logarithm of *p* value (-Lg*p*). The larger the -Lg*p* value, the smaller the *p* value and the higher the significance level of the target gene signal pathway. **(C)** MicroRNA-Gene network diagram of miR-19b-3p. The blue square in the figure refers to miR-19b-3p, the red circle refers to target genes, and the green arrow refers to the signal pathways involved in the target gene. Lines represent the regulatory relationship between miR-19b-3p and target genes.

Based on the GO and KEGG signal pathway analyses, we first predicted mRNAs that could be regulated by miR-19b-3p. Then we performed an enrichment analysis to infer the putative biological pathways that might be involved in the miRNA regulation. We detected 288 mRNAs regulated by miR-19b-3p (see **Supplementary Data 4**). Significant gene functions and pathways putatively altered in UD and BD patients were selected based on the standards of *p*<0.01 (GO) and *p*<0.05 (KEGG). GO results revealed that most functions associated with miR-19b-3p regulation were related to signal transduction, neuron growth, cell differentiation, and apoptosis (**Figure 3A**). The most relevant KEGG signal pathways were involved in enrichment of biological processes, which included mTOR, autophagy, FoxO, prolactin, p53, and the PI3-K/Akt signaling pathway (**Figure 3B**). A more intuitive miRNA-mRNA-gene network diagram was produced to investigate the potential role of miR-19b-3p in the pathogenesis of depression (**Figure 3C**). The most relevant target genes were MAPK1, PTEN, TGFBR2, PRKAA1, PIK3R3, and RAF1, which were involved in multiple pathways.

### Correlation Between miRNA Expression and Stress-Related Psychological Variables

A binary logistic regression model was produced to examine the correlation between miR-19b-3p expression and exposure to childhood trauma in patients with UD and BD, with adjustments for demographic variables. The results showed that the expression level of miR-19b-3p (OR=5.717, 95%CI:1.497–21.835, *p*=0.011) and the overall severity of childhood trauma (OR=1.099, 95%CI:1.012-1.192, *p*=0.025) were significantly associated with greater risk of BD. Among the sub-types of childhood trauma (emotional abuse, physical abuse, sexual abuse, emotional neglect, and physical neglect), there was a weak association between physical neglect and UD (OR=0.773, 95%CI:0.598-0.999, *p*=0.049) (**Table 2**). The other specific types of childhood trauma were not significantly correlated with any morbid status.

**Table 2:** The logistic regression of miR-19b-3p and childhood trauma characteristics between the patients with UD and BD.

	B	*p* value	OR	95%CI	
miR-19b-3p	1.743	0.011*	5.717	1.497	21.835
Total score of CTQ	0.094	0.025*	1.099	1.012	1.192
Physical neglect	−0.258	0.049*	0.773	0.598	0.999

UD, unipolar depression; BD, bipolar depression; CTQ, childhood trauma questionnaire. Physical neglect: a subtype of childhood trauma. *p < 0.05

## Discussion

In the present study, we systematically investigated the genome-wide miRNA expression profile in plasma from patients with UD and BD as compared to healthy subjects. Among 42 differentially expressed miRNAs, three miRNAs (miR-19b-3p, miR-3921, and miR-1180-3p) were selected to validate the microarray chip results. The novel miR-3921 was significantly up-regulated in the UD group but was not verified. miR-1180-3p was up-regulated in UD and BD patients but also was not verified. To date, no published reports have associated these two miRNAs with psychiatric disorders. However, miR-19b-3p was significantly down-regulated in the patient group, which indicates that peripheral miRNAs are possible non-invasive biomarkers with the necessary diagnostic accuracy. Furthermore, a specific miRNA related to psychological factors might help differentiate BD from UD. The BD patients were more likely to exhibit the combination of over-expressed miR-19b-3p and more severe childhood trauma when UD and BD patients were compared.

### miR-19b-3p and Its Pathology in Psychiatric Diseases

MiR-19b-3p belongs to the miR-17/92 cluster, which exerts powerful effects on lymphocyte development, proliferation, activation, differentiation, and cytokine production (40, 41). Gantier et al. first reported that miR-19b regulated the activity of nuclear factor-κB (NF-κB) signaling in inflammation (42). Over-expression of miR-19b-3p inhibited the production of IL-6 and IL-8, and the interaction of miR-19b-3p with its direct target gene, G protein-coupled receptor kinase 6 (GRK6), was discovered to affect inflammation (43).

TNFAIP3, which is negatively regulated by miR-19b-3p, is widely recognized as an important regulator of inflammation (44). An intriguing role for miR-19b-3p is to regulate the neuroinflammatory response induced by the Japanese encephalitis virus (JEV) *via* enhancement of NF-κB signaling (45). Dwivedi et al. reported that miR-19b was over-expressed in the prefrontal cortex of rats with stress-induced depression with chronic administration of exogenous corticosterone (46). Although the epigenetic mechanisms of miR-19b-3p in psychiatric disorders remain unclear, various studies point to the effects of this miRNA in the communication between the immune system and the brain, which is known as a new area in psychiatry, specifically, immunopsychiatry.

### The Target Genes of miR-19b-3p in Unipolar Depression and Bipolar Disorder

Our in silico results predicted a series of target genes and biological pathways for miR-19b-3p. Since patients with major depression who experienced childhood trauma were susceptible to immune dysregulation, we first check the biological processes associated with miR-19b-3p in immunomodulation between UD patients and BD patients. GO analysis predicted that the target gene function of miR-19b-3p, was enriched in the Wnt signaling pathway. It is consistent with a previous study that dysregulated expression of Wnt-related genes was shown in BD (47). Numerous studies have shown that suppression of Wnt signaling could induce both manic and depressive behaviors and exacerbate a proinflammatory state leading to increased neuronal apoptosis (48). In our study, the Wnt pathway was the most significantly enriched biological function, indicating its potential role in identifying UD and BD patients.

KEGG enrichment analyses predicted that the most significant target gene pathway was the mammalian rapamycin (mTOR) signaling pathway. Accumulating evidences suggested that mTOR signaling was dysregulated in depression (49). Activated mTOR signaling might be related to antidepressant-like effects in the hippocampus by modulating inflammatory cytokines such as interleukin-1β (IL-1β), interleukin-6 (IL-6), and tumor necrosis factor-α (TNF-α) (50). Autophagy signaling was the second, riched pathway regulated by miR-19b-3p. Dysregulation of autophagy leads to various disease manifestations, such as inflammation, metabolic alterations, and neurodegeneration (51). Some antidepressants induce inflammatory suppression through decreased serum levels of IL-1β and IL-18 and decreased NLRP3 protein expression *via* the autophagy pathway (52). Recent findings indicated that the forkhead box O (FoxO) signaling pathway is involved in the development of major depression and constitutes a potential therapeutic target in the treatment of depression (53). Although there is no evidence for FoxO involvement in BD, our results suggested that this pathway played an important role *via* miR-19b-3p in both UD and BD.

The prolactin pathway was identified in the differentiation between UD and BD. Hyperprolactinemia is reported commonly in subjects with a psychotic disorder which could due to stress, while information regarding mood disorder patients was particularly lacking (54). The p53 signaling, identified differently between UD and BD patients in our study, has not been reported to be associated with psychiatric disorders previously. Although the phosphatidylinositol 3-kinase (PI3K)-Akt signaling pathway was less strongly associated with miR-19b-3p expression, it contains the largest number of target genes. PI3K-Akt signaling is involved in the inhibition of the inflammatory response of lipoteichoic acid-stimulated macrophages (55). Reports indicate that some antidepressants used in clinical practice exert therapeutic efficacy *via* the promotion of the hippocampal PI3k-Akt-mTOR signaling pathway (56).

Mitogen-activated protein kinase 1 (MAPK1) had the highest predictive value among all the target genes of miR-19b-3p. Emerging evidence has revealed that alteration of MAPK1 is associated with psychiatric disorders, such as major depressive disorders, bipolar disorder and schizophrenia (57, 58). MAPK1 is highly expressed in the prefrontal cortex and hippocampus, and can modulate neuronal growth and differentiation, synaptic plasticity, and inflammatory processes *via* mTOR, FoxO, and other signaling pathways (59, 60).

Phosphatase and tensin homolog (PTEN) is a significant target of miR-19b-3p, and it directly regulates the PI3-K-Akt-mTOR signaling pathway, which is regarded as the most critical pathway for many neurobiological functions in the brain. PTEN regulates neuron cell size and affects dendritic growth, and it also acts as a significant tumor suppressor gene through the modulation of the inflammatory process (61, 62). Altered expression of PTEN in the blood is considered a biomarker for suicidal tendencies (63), as well as in the prefrontal cortex and hippocampus of suicide victims (64). The possibility of using miR-19b-3p and its target genes, such as PTEN to identify stress-related neuropathology in mood disorders, is encouraging. An upstream molecule in the mTOR pathway (PRKAA1) was down-regulated by miR-181a and facilitated hippocampal fear memory consolidation (65). Fear inhibition is related to trauma events (66). Therefore, PRKAA1 is one of significant target genes of miR-19b-3p that could be associated with stress-induced bipolar patients *via* activation of the mTOR pathway.

### Childhood Traumatic Exposure and miRNA Alterations in Depressive Patients

Early life adversities, such as emotional, physical, and sexual abuse or neglect in childhood, are associated with poor psychological health outcomes in adults. Childhood traumatic experiences play an important role in developing BD, induction of more severe clinical symptoms, impairing emotion regulation and cognitive function, and lead to a much higher risk for suicide (28). Recent studies have explored the epigenetic mechanisms between childhood trauma and major depression, as well as schizophrenia (14). Our findings provide a significant association between childhood traumatic experiences and altered expression of miR-19b-3p in BD. Based on our bioanalysis results, plasma miR-19b-3p might be a biomarker associated with the impact of childhood trauma on UD and BD through the involvement of inflammatory processes.

Increased inflammation has been described in healthy individuals exposed to childhood trauma, suggesting a potentially causal role in the future onset of depression (67, 68). The Dwivedi group focused on epigenetic mechanisms of depression and suicide and recently reported that proinflammatory cytokines (e.g., TNF-α) and miR-19a-3b were up-regulated in the dorsolateral prefrontal cortex (DLPFC) in suicide victims, and both molecules were increased in the peripheral blood mononuclear cells of depressed patients with severe suicidal ideation (69).

Another important finding showed that unipolar patients with the lowest expression of peripheral miR-19p-3b among the three groups experienced more physical neglect, which has not been reported previously. The effects of neglect can be as traumatic as or even more traumatic than the effects of abuse, and the traumatic effects persist into adulthood (70). Neglect has several forms. Physical neglect could prompt the individual to develop psychological unavailability, which impacts the development of depressive symptoms. Further research is needed on possible epigenetic mechanisms of neglect associated with miRNAs.

### Strengths and Limitations

This study has some strengths. First, we stringently recruited subjects in the screening phase. There were nearly identical clinical manifestations of the unipolar and bipolar patients, which could exclude possible confounding caused by distinct profiling between the two groups. Second, the differentiated miRNAs were systematically screened through the whole miRNome profiling on unipolar and bipolar depressive patients and healthy controls and was validated in a larger independent sample to increase the reliability of the results. Third, we provided an in-depth discussion of the possible miRNA targets and the integration of potential molecular mechanisms with environmental factors. We explored the possible use of peripheral miRNAs as non-invasive diagnostic biomarkers of mood disorders from an immune-psychiatry perspective and prompted further research on the role of miR-19p-3b in depression.

Nevertheless, the relationship of miRNA expression in plasma and the brain is not clear, since we only observed alterations in peripheral miRNAs. It is a concern that there were two selected miRNAs eliminated in the validation phase, which might be due to the limited sample size. A miRNA panel would be a more robust method of diagnosis of UD or BD. We did not examine any circulating inflammatory factors that were potentially related to the disorders. Therefore, the association between miRNA expression and immunological regulation remains unclear. A well-designed study would need to be conducted to develop a psychoneuroimmunology network of childhood trauma, immunological indices, altered miRNAs, and the disorder.

## Conclusions

This study showed that the expression profiling of plasma miRNAs was altered both in UD and BD. miR-19b-3p was down-regulated in patients with depression and was validated to be over-expressed in BD but not UD. The target genes for miR-19b-3p were primarily enriched in the Wnt signaling and mTOR pathways. These miRNA gene targets elucidate the pathways and mechanisms involved in neuroimmunology pathways and depression pathogenesis.

## Data Availability Statement

All data have been submitted to the GEO repository (code number: GSE152267).

## Ethics Statement

The studies involving human participants were reviewed and approved by Affiliated Brain Hospital of Nanjing Medical University ethics committee. The patients/participants provided their written informed consent to participate in this study.

## Author Contributions

YC: collected data, conducted the statistical analysis, drafted the manuscript, edited, and submitted the manuscript. JS, HL, QW, XC, HT, RY: collected data, reviewed, and revised the manuscript. QL: statistical analysis, critically reviewed, edited, and revised the manuscript. ZY: conceptualized and designed the study, critically reviewed, and revised the manuscript.

## Funding

This work was supported by National Key R&D Program of China under grant number 2018YFC1314600; The National Natural Science Foundation of China under grant number 81871066,81571639; Jiangsu Provincial Medical Innovation Team of the Project of Invigorating Health Care through Science, Technology and Education under grant number CXTDC2016004; Jiangsu Provincial key research and development program under grant number BE2018609; Jiangsu Provincial Medical Youth Talent-The Project of Invigorating Health Care through Science, Technology and Education, QNRC2016049; The Science and Technology Program of Nanjing, 201803030.

## Conflict of Interest

The authors declare that the research was conducted in the absence of any commercial or financial relationships that could be construed as a potential conflict of interest.
