# Supplementary material for: Plasma microRNA Array Analysis Identifies Overexpressed miR-19b-3p as a Biomarker of Bipolar Depression Distinguishing From Unipolar Depression
Source: Front Psychiatry. 2020 Aug 11;11:757. doi: 10.3389/fpsyt.2020.00757 (PMC7432143; doi:10.3389/fpsyt.2020.00757)
Supplement: Supplementary file 1 [file DataSheet_1.docx]

**Plasma microRNA array analysis identifies overexpressed miR-19b-3p as a biomarker of bipolar depression distinguishing from unipolar depression**

*Yu Chen^a^, Jiabo Shi^a^, Haiyan Liu^a^, Qiang Wang^e^, Xiangxiang Chen^a^, Hao Tang^a^, Rui Yan^a^, Qing Lu^b,c*^ , Zhijian Yao^a,d*^*

Supplemental data 1. Amplification primer

| **Name** | **Sequence** |
| --- | --- |
| **U6-F** | CTCGCTTCGGCAGCACA |
| **U6-R** | AACGCTTCACGAATTTGCGT |
| **hsa-miR-3921-F** | GCGCGTCTCTGAGTACCATATG |
| **hsa-miR-3921-RT** | GTCGTATCCAGTGCAGGGTCCGAGGTATTCGCACTGGATACGACACAAGG |
| **hsa-miR-1180-3p-F** | TTTCCGGCTCGCGTGG |
| **hsa-miR-1180-3p-RT** | GTCGTATCCAGTGCAGGGTCCGAGGTATTCGCACTGGATACGACACACAC |
| **hsa-miR-19b-3p-F** | CGTGTGCAAATCCATGCAA |
| **hsa-miR-19b-3p-RT** | GTCGTATCCAGTGCAGGGTCCGAGGTATTCGCACTGGATACGACTCAGTT |
| **Reverse Primer** | AGTGCAGGGTCCGAGGTATT |

Supplementary data 2.

**Demographic and clinical characteristics of the subjects for miRNA array screening**

| **variables** | **The subjects for miRNA profiling screening** | | | **Significance** | |
| --- | --- | --- | --- | --- | --- |
|  | **UD(n=7)**  **Mean (SD)** | **BD(n=7)**  **Mean (SD)** | **HC(n=6)**  **Mean (SD)** | ***p*(two groups)** | ***p*(three groups)** |
| **Age (years)** | 37.57±6.90 | 33.57±8.87 | 37.50±5.99 | 0.365 | 0.535 |
| **Female** | 3 (42.9%) | 3 (42.9%) | 3 (50.0%) | 1.000 | 0.958 |
| **Male** | 4 (57.1%) | 4 (57.1%) | 3 (50.0%) |  |  |
| **Education (years)** | 14.71±2.56 | 14.00±1.92 | 13.00±1.55 | 0.957 | 0.355 |
| **Duration of illness (months)** | 16.57±15.92 | 52.00±58.70 |  | 0.149 |  |
| **Total score of HAM-D_24_** | 33.29±4.96 | 28.00±6.11 |  | 0.101 |  |
| **Subscore of HAM-D_24_** | | | | | |
| Anxiety/somatization | 9.43±2.94 | 5.71±2.75 |  | 0.031* |  |
| Cognitive disturbance | 4.29±2.22 | 4.29±2.14 |  | 1.000 |  |
| Retardation | 8.86±1.07 | 7.71±1.98 |  | 0.203 |  |
| Hopelessness | 5.57±1.51 | 5.29±1.80 |  | 0.753 |  |
| Sleep disturbance | 3.57±1.40 | 4.00±1.41 |  | 0.579 |  |
| Weight loss | 0.14±0.38 | 0.43±0.79 |  | 0.064 |  |
| Circadian fluctuation | 0.33±0.61 | 0.26±0.51 |  | 0.403 |  |
| **Total score of CTQ** | 47.14±11.19 | 56.00±17.72 |  | 0.285 |  |
| **Subscore of CTQ** | | | | | |
| Emotional abuse | 8.00±4.76 | 11.86±7.11 |  | 0.256 |  |
| Physical abuse | 5.57±1.13 | 7.71±3.73 |  | 0.171 |  |
| Sexual abuse | 5.57±1.13 | 5.00±0.00 |  | 0.207 |  |
| Emotional neglect | 12.00±5.26 | 14.00±6.58 |  | 0.542 |  |
| Physical neglect | 8.71±4.19 | 10.86±5.52 |  | 0.429 |  |

UD: unipolar depression; BD: bipolar depression; HC: healthy controls; *p<0.05.

Supplementary data 3

**Differential expression of miRNAs between healthy groups and patients with unipolar depression and bipolar depression**

| miRNA | UD (FC) | BD (FC) | HC (FC) | *p* | Style |
| --- | --- | --- | --- | --- | --- |
| hsa-miR-1180-3p | 1.793 | 2.489 | 1.053 | 0.000 | up |
| hsa-miR-1281 | 5.531 | 5.088 | 4.187 | 0.200 | up |
| hsa-miR-1298-3p | 2.990 | 1.779 | 0.909 | 0.282 | up |
| hsa-miR-297 | 1.948 | 2.342 | 0.911 | 0.042 | up |
| hsa-miR-3148 | 1.407 | 1.640 | 0.748 | 0.041 | up |
| hsa-miR-337-3p | 2.760 | 2.272 | 1.029 | 0.011 | up |
| hsa-miR-3619-5p | 4.004 | 2.562 | 2.207 | 0.667 | up |
| hsa-miR-378h | 4.576 | 3.525 | 2.273 | 0.105 | up |
| hsa-miR-3910 | 2.030 | 1.473 | 0.678 | 0.134 | up |
| hsa-miR-3921 | 5.110 | 4.136 | 2.427 | 0.035 | up |
| hsa-miR-4706 | 4.618 | 3.520 | 2.692 | 0.249 | up |
| hsa-miR-5094 | 1.820 | 1.450 | 0.793 | 0.187 | up |
| hsa-miR-548aa | 1.433 | 1.199 | 0.746 | 0.131 | up |
| hsa-miR-548ap-3p | 1.559 | 1.274 | 0.765 | 0.179 | up |
| hsa-miR-548t-3p | 1.432 | 1.197 | 0.745 | 0.132 | up |
| hsa-miR-570-3p | 1.399 | 0.896 | 0.572 | 0.392 | up |
| hsa-miR-628-5p | 2.377 | 1.641 | 0.927 | 0.205 | up |
| hsa-miR-6511b-5p | 3.972 | 2.936 | 1.976 | 0.129 | up |
| hsa-miR-668-5p | 3.553 | 2.552 | 2.189 | 0.531 | up |
| hsa-miR-6732-5p | 8.281 | 7.494 | 7.145 | 0.427 | up |
| hsa-miR-6750-5p | 3.960 | 3.040 | 2.151 | 0.170 | up |
| hsa-miR-6794-5p | 4.983 | 4.438 | 3.920 | 0.184 | up |
| hsa-miR-6800-3p | 4.125 | 3.584 | 2.642 | 0.146 | up |
| hsa-miR-8075 | 7.500 | 6.679 | 6.158 | 0.363 | up |
| hsa-miR-877-5p | 3.602 | 2.833 | 2.219 | 0.267 | up |
| hsa-miR-106a-5p | 2.561 | 5.773 | 5.713 | 0.844 | down |
| hsa-miR-106b-5p | 1.208 | 2.920 | 4.084 | 0.294 | down |
| hsa-miR-130b-3p | 2.028 | 2.325 | 3.098 | 0.171 | down |
| hsa-miR-140-3p | 2.664 | 4.110 | 5.221 | 0.238 | down |
| hsa-miR-150-5p | 1.320 | 4.119 | 4.007 | 0.784 | down |
| hsa-miR-16-5p | 3.603 | 7.757 | 7.517 | 0.750 | down |
| hsa-miR-181a-5p | 1.643 | 3.574 | 4.639 | 0.343 | down |
| hsa-miR-19b-3p | 3.501 | 4.259 | 6.272 | 0.019 | down |
| hsa-miR-22-3p | 2.953 | 4.030 | 6.345 | 0.051 | down |
| hsa-miR-378a-3p | 1.582 | 2.553 | 3.719 | 0.114 | down |
| hsa-miR-378c | 0.665 | 1.129 | 1.671 | 0.085 | down |
| hsa-miR-3937 | 1.409 | 1.274 | 2.146 | 0.042 | down |
| hsa-miR-423-3p | 0.896 | 2.246 | 2.694 | 0.536 | down |
| hsa-miR-425-5p | 1.646 | 5.059 | 5.358 | 0.931 | down |
| hsa-miR-6813-5p | 1.264 | 1.043 | 1.965 | 0.024 | down |
| hsa-miR-7641 | 0.992 | 1.967 | 3.611 | 0.115 | down |
| hsa-miR-93-5p | 2.653 | 6.362 | 6.494 | 0.954 | down |

UD: unipolar depression; BD: bipolar depression; HC: healthy controls; FC: fold change;style: up: overexpression; style: down: downexpression.

Supplemental data 4.  **The 288 predicted target mRNAs of has-miR-19b-3p:**

| NR3C2  BACE1  PTEN  ATXN1  HIPK3  ARID4B  MYLIP  ESR1  NCOA3  KAT2B  SOCS1  BCL2L11  TGFBR2  CUL5  TLR2  PRKAA1  PPP2R5E  ZMYND11  RPS4Y1  PRRG4  LCLAT1  SKIL  BAMBI  SLC38A2  DEF8  RNF167  BCL3  EVI5L  SLC6A8  KCNJ2  ASNA1  MB21D1  PTPRB  TNFRSF10B  ZNF800  YY1 | MCC  JAZF1  HNRNPF  GNPTAB  FRS2  DYNC1LI2  DEPDC1  DBN1  CFL2  GDNF  ARL8A  AGO3  ZNF680  ZNF107  PABPC4L  C15orf38-AP3S2  CLVS2  CCDC80  ZNF544  ZDHHC7  RACGAP1  FGFR1OP  HOMER1  ODF4  MBD3  MTX3  PTCD2  ETV3  C6orf132  DIP2A  MTHFD1  UBL3  KIAA0907  TMEM2  KITLG  FKBP15 | PHLDA3  NRBF2  MAPK1  CAMSAP2  ZNF154  SLC46A1  UBE2D3  SESN3  SATB1  NUP54  LZIC  BEND3  PALM2-AKAP2  AKAP2  USP8  RAN  PPTC7  PFN2  MRPL17  DCC  ZNF134  VPS37B  RBM20  GRB10  DDX3X  RRAGD  CEP170  MALT1  HADHB  TMEM64  SLC12A7  PFN1  DGKH  CLOCK  ADSS  ZNF367 | MTMR12  MKL2  MEF2A  MCM3AP-AS1  MBD4  MAP3K14  LOC613266  LIN9  KLHL3  KIF3A  CCSER2  ATG14  INO80  HNRNPU  HIC1  HECW2  HDAC4  GRSF1  GPAM  GAK  FOXP1  FBXO48  FBXO10  MIGA1  DENND6A  FAM102A  EXOC7  EPS15  EPN2  EIF4E2  EHD1  DUT  DSCR3  CIT  CBX7  CBX5 | WNT7B  THBS1  TGOLN2  STX6  SGK1  RHOB  PPP6R1  NACC1  MTMR6  MAP3K9  KLHL11  KIF13A  IMPDH1P11  FBLIM1  EOGT  ELL2  DLG5  CD164  CCNA2  CAPRIN2  ANKRD50  ACTB  ABHD14B  YTHDF1  ZFYVE26  FOXQ1  DICER1  ARPP19  WDR45B  SLC48A1  MMGT1  BRWD3  NICN1  PTBP2  ZMAT3  TRIM37 | TMTC1  XIAP  WNT10A  WDR1  VAMP1  TRAK2  TP53INP1  TNPO2  TNIP1  TNFAIP3  TFB1M  STK4  SOCS3  SNX17  SLC9A6  SLC25A12  SH3KBP1  SERBP1  SEPHS2  SEL1L  SEC63  SBF2  S1PR2  RNF216  RGL1  RAPGEF6  RAF1  RAB2B  PIK3R3  PIGS  PGM2L1  PGK1  PCDH10  PRKN  OTUD7B  NAPB | TNFRSF11A  TMEM117  RPF2  NDRG1  MFF  STOX2  SNX5  RLIM  SFTPA1  MBNL3  GIGYF1  GFPT1  DCUN1D3  CREBRF  CERCAM  CHEK2  CASZ1  BMP3  ARC  ACSL4  ACBD5  ZNF138  MAVS  RBM38  TMEM138  DAD1  WNK3  WASL  UBE2A  TXLNG  SOX6  RAP1A  PURG  PRICKLE2  MPRIP  MECP2 | IDNK  C2orf42  PRR14L  SDE2  ARPIN  SPTSSA  GSKIP  BTBD7  BRD9  BCL7B  B4GALT1  AZIN1  ATXN7  ATPAF1  ATG5  ATG2B  ARMC8  ARAP2  ANGEL2  AHDC1  PTENP1  PKNOX1  MTUS1  PITX1  CSNK2A1  DCBLD2  EREG  PHLDA1  PKM  B3GALNT2  DSEL  IFITM1  TPRG1L  ZNF423  RPAP2  ZNF618 |
| --- | --- | --- | --- | --- | --- | --- | --- |
